# The Role of the Physician in Time of War

**Published:** 1914-10-17

**Authors:** 


### The Hospital

The Workers' Newspaper of Administrative Medicine and Institutional Life, Administration, National Insurance and Health.

No. 1478, Vol. LVII. SATURDAY, OCTOBER 17, 1914. PRICE ONE PENNY.

### THE ROLE OF THE PHYSICIAN IN TIME OF WAR.

The civic and national obligation for service applies to the medical profession as it applies to other citizens, and nowhere, we are sure, will its justness be more readily and resolutely recognised. In active service, and exposed to all the dangers of the battlefield and to the perils of naval warfare, we shall not fail to see our *confrères* engaged at the post of duty and bringing succour to those who are ready to perish. With equal obligation, though in less acute circumstances, there will be instructed minds and skilful hands prompt to place at the service of our sick and wounded soldiers all the resources of modern medical and surgical science.

In the public judgment, and not without some warrant, it is the practice of surgery rather than that of medicine which seems to be more particularly under demand when the mischiefs of war begin to display themselves. Wounds and other injuries are the certain consequences of fighting, and are the most obvious and dramatic results of warfare. They catch the public eye and impress the public mind, and for their relief and repair expectation turns with confidence to the surgeon. To his skill and judgment we are all anxious to pay a full tribute, and we may allow that he stands in the forefront of professional opportunity. To some extent all this seems to mean the eclipse of the physician, and it may, therefore, be well, at the present moment, to recall how necessary is the latter, if reasonable and adequate succour is to be afforded to those who have to sustain the immediate strain and shock of war.

In the first place, it must be remembered that all the casualties of warfare are not mechanical or susceptible of relief by surgical art. Amidst masses of men, numbered by hundreds of thousands, even though the individuals represent the picked manhood of the nation, there must needs be some proportion of sickness and disease. There are circumstances, inevitable in every campaign, which contribute to this end. The physical and mental strain inflicted on an army in the field is tremendous, and in the present day, when men have become more sensitive and instruments of warfare more terrible, it is little wonder that individuals collapse under it. Supplies of food and water, sufficient in quantity and quality, cannot be guaranteed by any foresight to millions of men liable to rapid and unexpected movements in a large theatre of war. Add to these influences the necessities of a rough and ready system of sanitation, the risks incident to exposure to unhappy weather conditions—and the need for the provision of aid and skill to relieve the victims of disease becomes evident. It is, of course, obvious that these victims have a not less decided claim than have those who fall under the bullet and the sword. For all alike there must be provided such help as medicine and surgery may afford. This aid is due to the individual both as a soldier and as a sick man. It has, however, a wider range. Men in hospital are no help, they are indeed a hindrance, to the active operations of warfare. What is wanted is that, in so far as possible, they shall be restored to efficiency, and shall be able to rejoin their fighting comrades. Hence, looked at even from the most material standpoint, it is profitable to secure for an army in the field not only efficient surgical but also efficient medical assistance.

Once more, modern experience has shown that in many cases bullet wounds of the trunk and even of the skull do better without active interference, rather than with it. Therefore, what such patients require is not the dexterity of the surgeon, but the judgment and art of the physician, who, by his general care, and even in some measure by medicinal treatment, may assist the restorative influences of Nature. Very much the same may be said of the position of many cases subsequent to operation. Their movement towards convalescence is a matter for skilled care and oversight when once the surgeon has performed his task. These considerations are of importance not only in relation to the individual patients. The strain on the surgeons and the pressure on the surgical accommodation during an active campaign are apt to be enormous, and obviously some relief would be afforded in each of these directions were patients as soon as possible removed from the responsibility of the surgeon and placed under medical care. One further point may be mentioned. It is that while in the rough processes of war professional refinements are hardly possible, there are few surgeons who would not in many individual experiences be glad to have the help and counsel of an experienced and capable medical colleague. We have made no allusion here to the large part played by medicine in the preservation of the health of an army by skilled advice on the selection of food, water supplies, camping sites, and other details included in the art of sanitation. What we have had before us is rather the actual casualties of the campaign, and our aim has been to show that in meeting these the learning and art of the physician and the technique of the surgeon must act in loyal harmony and co-operation.

